# The genome sequence of the Tropical Bluetail Damselfly, *Ischnura senegalensis *(Rambur, 1842)

**DOI:** 10.12688/wellcomeopenres.23747.1

**Published:** 2025-02-24

**Authors:** Beatriz Willink, Wellcome Sanger Institute Tree of Life Management, Samples and Laboratory team, Wellcome Sanger Institute Scientific Operations: Sequencing Operations, Wellcome Sanger Institute Tree of Life Core Informatics team, Tree of Life Core Informatics collective

**Affiliations:** 1 Department of Biological Science, National University of Singapore, Singapore 117558, Singapore; 2 Department of Entomology, Cornell University, Ithaca, New York, USA

**Keywords:** Ischnura senegalensis, Tropical Bluetail Damselfly, genome sequence, chromosomal, Odonata

## Abstract

We present a genome assembly from a specimen of *Ischnura senegalensis* (Tropical Bluetail; Arthropoda; Insecta; Odonata; Coenagrionidae). The assembly contains two haplotypes with total lengths of 1,599.82 megabases and 1,602.78 megabases. Most of haplotype 1 (96.41%) is scaffolded into 14 chromosomal pseudomolecules, including the X sex chromosome, which haplotype 2 is a scaffold-level assembly. The mitochondrial genome has also been assembled and is 18.11 kilobases in length.

## Species taxonomy

Eukaryota; Opisthokonta; Metazoa; Eumetazoa; Bilateria; Protostomia; Ecdysozoa; Panarthropoda; Arthropoda; Mandibulata; Pancrustacea; Hexapoda; Insecta; Dicondylia; Pterygota; Palaeoptera; Odonata; Zygoptera; Coenagrionoidea; Coenagrionidae; *Ischnura*; *Ischnura senegalensis* (Rambur, 1842) (NCBI:txid126660)

## Background

**Figure 1.**
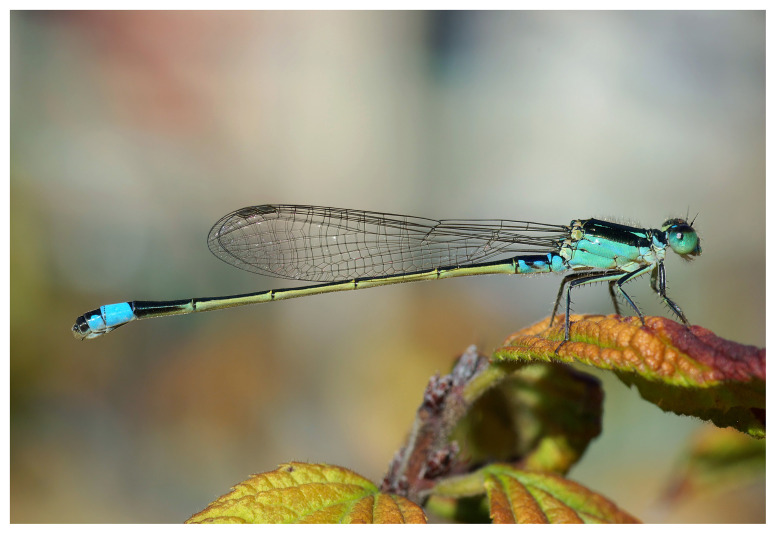
Photograph of *Ischnura senegalensis* (not the specimen used for genome sequencing). Image by Laitche.

The Tropical Bluetail damselfly ( *Ischnura senegalensis*) ( Figure 1) is one of the most widely distributed species of pond damselflies (family Coenagrionidae) (Willink *et al*., 2024b). Its geographic range extends from sub-Saharan Africa to Japan, including populations across South and South-east Asia (GBIF Secretariat, 2024). Mitochondrial data suggest that Asian populations are interconnected by gene flow, while African and Asian populations are highly differentiated (Jiang *et al*., 2023). On par with its vast geographic range, the Tropical Bluetail damselfly breeds in a wide diversity of aquatic habitats (Sharma & Clausnitzer, 2016) and is relatively tolerant to habitat disturbances (Badu *et al*., 2024; Samways & Grant, 2008). Thus, the Tropical Bluetail damselfly is assessed as Least Concern by the IUCN Red List of Threatened Species (Sharma & Clausnitzer, 2016).

As many of its congeners, *I. senegalensis* comprises a female-limited colour polymorphism (Blow *et al*., 2021). One of the two female morphs in this species has a black thoracic background with green-blue markings and a bright-blue patch on its eighth abdominal segment. This morph is often referred to as *andromorph* because its colour pattern is very similar to that of males. The other female morph, known as *gynomorph*, undergoes drastic colour changes during adult development. While sexually immature, *gynomorph* females exhibit a bright-orange thoracic colouration. This colouration darkens over the course of reproductive development, with mature gynomorph females displaying a green-brown thorax (Takahashi *et al*., 2012).

Both the developmental colour changes and the final colour differences between morphs influence the extent of male mating and pre-mating harassment, which typically have negative consequences for female fitness (Takahashi & Watanabe, 2009; Takahashi & Watanabe, 2011). In short, the bright colouration of immature *gynomorph* females acts as a signal of reproductive unsuitability, and prevents male harassment (Takahashi *et al*., 2012; Takahashi & Watanabe, 2011). However, when mature *gynomorph* females occur at high frequency, they experience increased male-mating attempts, resulting in reduced food intake and reduced fecundity (Takahashi & Watanabe, 2009; Takahashi & Watanabe, 2010). Because male mating preferences change plastically with shifting female morph frequencies (Takahashi & Watanabe, 2009), the ensuing rise in *andromorph* relative fitness is short lived, and the two female morphs are maintained in sympatry and oscillating in frequency (Takahashi *et al*., 2010). Interestingly, sexual conflict over mating rates and frequency-dependent male harassment prevents morph fixation across heterogeneous habitats, and even though equilibrium morph frequencies vary along ecological clines (Takahashi *et al*., 2011).

In recent years, the evolutionary history and molecular basis of morph differentiation in the Tropical Bluetail damselfly have been under active investigation. Comparative evidence suggests that the occurrence of *gynomorph* and *andromorph* females is a trans-species polymorphism in *Ischnura* damselflies (Blow *et al*., 2021). *Andromorph* females share a common origin in the Tropical Bluetail damselfly, the Common Bluetail damselfly ( *Ischnura elegans*), and likely, several other species in their clade (Blow *et al*., 2021; Willink *et al*., 2024a). Analyses based on the *I. elegans* genome assembly indicate that the *andromorph* allele evolved in association with a signature of duplicated inversions and an expansion of genomic sequences in the smallest autosomal chromosome (Willink *et al*., 2024a). Even though both the Tropical Bluetail and the Common Bluetail damselflies exhibit these genomic signatures, dominance hierarchies differ between species, with the *andromorph* allele being dominant in the Common Bluetail and its close relatives (Cordero, 1990), but recessive in the Tropical Bluetail damselfly (Takahashi *et al*., 2019).

In the Tropical Bluetail damselfly, the *andromorph* genotype is associated with differential expression of colour and sex determination genes. Particularly, the classic sex-determination gene *doublesex* ( *dsx*) is differentially spliced and expressed between *andromorph* and *gynomorph* females (Takahashi *et al*., 2019). As in many other insects (Geuverink & Beukeboom, 2014), *dsx* produces a short male-associated transcript and a longer female-associated transcript in *I. senegalensis* (Takahashi *et al*., 2019). Notably, *andromorph* females show reduced expression of the long *dsx* compared to *gynomorph* females, and their expression of the short *dsx* transcript is intermediate between males and *gynomorph* females (Takahashi *et al*., 2019). Experimentally silencing of the short *dsx* transcript in *andromorph* females restores *gynomorph* colouration, suggesting a function for *dsx* downstream of the morph determination locus, and involved in the development of the male-like colour pattern of *andromorphs* (Takahashi *et al*., 2021).

In addition to the study of female-limited morphs, the Tropical Bluetail damselfly has also become a model for molecular and developmental studies in Odonata. For example, studies using the Tropical Bluetail and the Common Bluetail damselfly have examined the structural mechanisms responsible for the production of blue colouration. This research shows that pteridine pigments, contained in densely packed light-scattering nanospheres, and photoluminescence emissions contribute to blue reflectance in males and *andromorph* females (Chuang *et al*., 2016; Henze *et al*., 2019; Okude & Futahashi, 2021). Similarly, a recent study on the evolution of the genetic regulation of insect metamorphosis, used the Tropical Bluetail damselfly to reveal a conserved function of *Krüppel homolog 1* and *E93* in the drastic morphological changes associated with adult development across the diverse clade of winged insects (Okude *et al*., 2022). Meanwhile, the gene *broad*, responsible for inducing pupation in holometabolous insects, was found to play a lineage-specific role in Odonata, maintaining nymph traits and repressing adult development (Okude *et al*., 2022).

Together, these studies showcase the ample potential of the Tropical Bluetail damselfly for research into the evolution, genetics, and development of phenotypic innovations in the ancient insect order Odonata. Such exciting avenues of future research critically rely on the development of genomic resources for this species. Here, we present a chromosome-level genome sequence for an *andromorph* female ( Figure 1) of the Tropical Bluetail damselfly ( *Ischnura senegalensis*), based on specimens collected at Clementi Forest, Singapore.

## Genome sequence report

### Sequencing data

The genome of a specimen of *Ischnura senegalensis* was sequenced using Pacific Biosciences single-molecule HiFi long reads, generating 49.71 Gb from 6.78 million reads. GenomeScope analysis of the PacBio HiFi data estimated the haploid genome size at 1,550.82 Mb, with a heterozygosity of 1.74% and repeat content of 22.47%. These values provide an initial assessment of genome complexity and the challenges anticipated during assembly. Based on this estimated genome size, the sequencing data provided approximately 31.0x coverage of the genome. Hi-C sequencing produced 98.59 Gb from 652.92 million reads. Table 1 summarises the specimen and sequencing information, including the BioProject, study name, BioSample numbers, and sequencing data for each technology.

**Table 1.** Specimen and sequencing data for *Ischnura senegalensis*.

Project information			
**Study title**	Ischnura senegalensis (tropical bluetail)		
**Umbrella BioProject**	PRJEB78898		
**Species**	*Ischnura senegalensis*		
**BioSpecimen**	SAMEA112696355		
**NCBI taxonomy ID**	126660		
Specimen information			
**Technology**	**ToLID**	**BioSample accession**	**Organism part**
**PacBio long read sequencing**	ioIscSene1	SAMEA112696364	whole organism
**Hi-C sequencing**	ioIscSene1	SAMEA112696364	whole organism
Sequencing information			
**Platform**	**Run accession**	**Read count**	**Base count (Gb)**
**Hi-C Illumina NovaSeq X**	ERR13494015	3.40e+08	51.34
**Hi-C Illumina NovaSeq X**	ERR13494016	3.13e+08	47.26
**PacBio Revio**	ERR13510307	2.57e+06	18.46
**PacBio Revio**	ERR13510308	4.21e+06	31.25

### Assembly statistics

The genome was assembled into two haplotypes using Hi-C phasing. Haplotype 1 was curated to chromosome level, while haplotype 2 was assembled to scaffold level. The assembly was improved by manual curation, which corrected 316 misjoins or missing joins and removed 1707 haplotypic duplications. These interventions reduced the total assembly length by 1.43%, decreased the scaffold count by 31.99%, and increased the scaffold N50 by 5.04%. The final assembly has a total length of 1,599.82 Mb in 964 scaffolds, with 5,153 gaps, and a scaffold N50 of 114.74 Mb ( Table 2).

**Table 2.** Genome assembly data for *Ischnura senegalensis*.

Genome assembly	Haplotype 1	Haplotype 2
Assembly name	ioIscSene1.hap1.1	ioIscSene1.hap2.1
Assembly accession	GCA_964251815.1	GCA_964247645.1
Assembly level	chromosome	scaffold
Span (Mb)	1,599.82	1,602.78
Number of contigs	6,117	6,510
Number of scaffolds	964	1,260
Assembly metrics (benchmark)	Haplotype 1	Haplotype 2
Contig N50 length (≥ 1 Mb)	0.46 Mb	0.45 Mb
Scaffold N50 length (= chromosome N50)	114.74 Mb	109.46 Mb
Consensus quality (QV) (≥ 40)	61.4	61.3
*k*-mer completeness	71.73%	71.86%
Combined *k*-mer completeness (≥ 95%)	99.19%	
BUSCO * (S > 90%; D < 5%)	C:97.0%[S:96.2%,D:0.8%], F:1.5%,M:1.5%,n:1,367	C:96.5%[S:95.4%,D:1.1%], F:1.8%,M:1.8%,n:1,367
Percentage of assembly mapped to chromosomes (≥ 90%)	96.41%	-
Sex chromosomes (localised homologous pairs)	X	-
Organelles (one complete allele)	Mitochondrial genome: 18.11 kb	-

* BUSCO scores based on the insecta_odb10 BUSCO set using version 5.5.0. C = complete [S = single copy, D = duplicated], F = fragmented, M = missing, n = number of orthologues in comparison.

**Figure 2.**
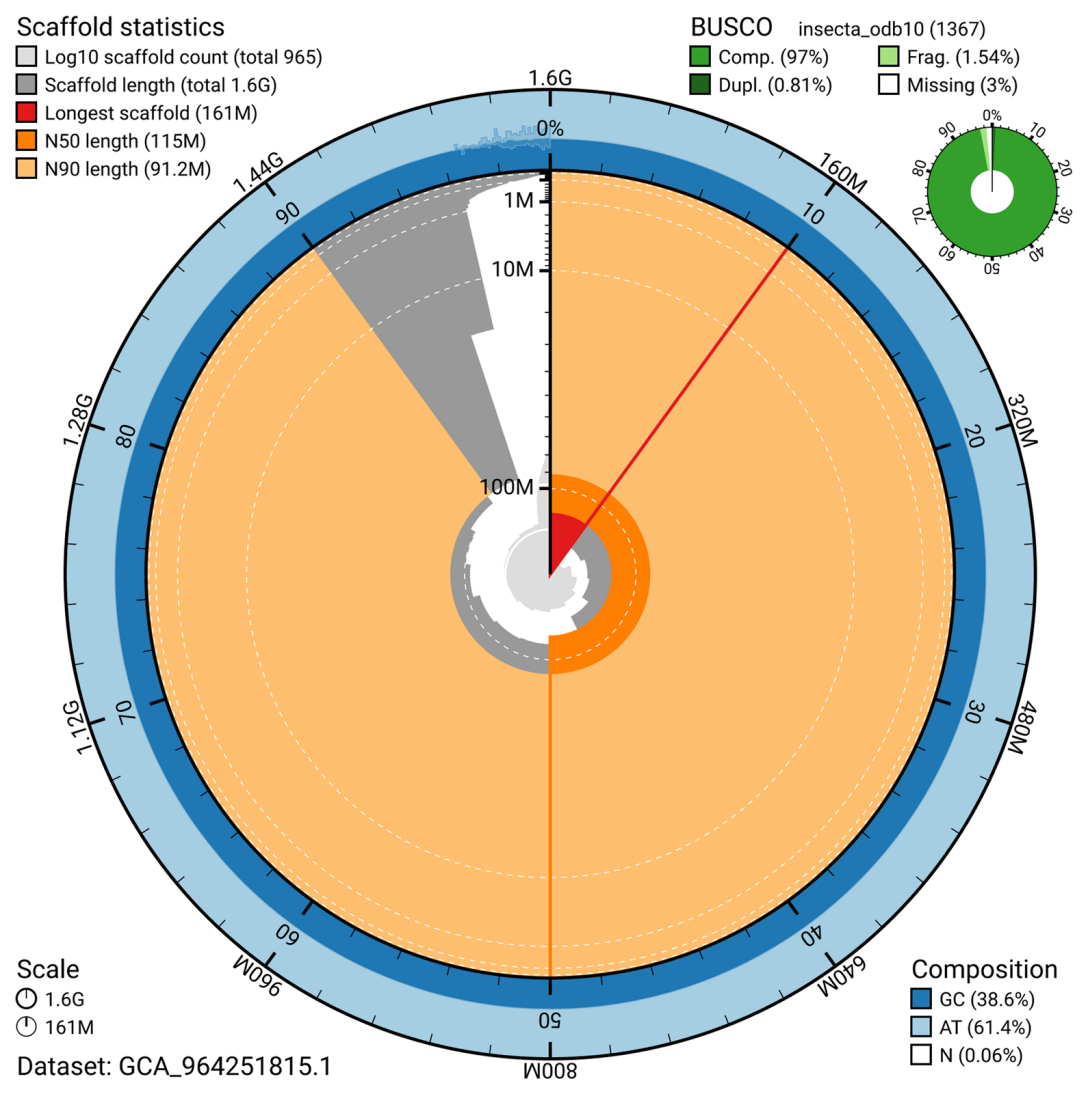
Genome assembly of *Ischnura senegalensis*, ioIscSene1.hap1.1: metrics. The BlobToolKit snail plot provides an overview of assembly metrics and BUSCO gene completeness. The circumference represents the length of the whole genome sequence, and the main plot is divided into 1,000 bins around the circumference. The outermost blue tracks display the distribution of GC, AT, and N percentages across the bins. Scaffolds are arranged clockwise from longest to shortest and are depicted in dark grey. The longest scaffold is indicated by the red arc, and the deeper orange and pale orange arcs represent the N50 and N90 lengths. A light grey spiral at the centre shows the cumulative scaffold count on a logarithmic scale. A summary of complete, fragmented, duplicated, and missing BUSCO genes in the insecta_odb10 set is presented at the top right. An interactive version of this figure is available at https://blobtoolkit.genomehubs.org/view/GCA_964251815.1/snail.

**Figure 3.**
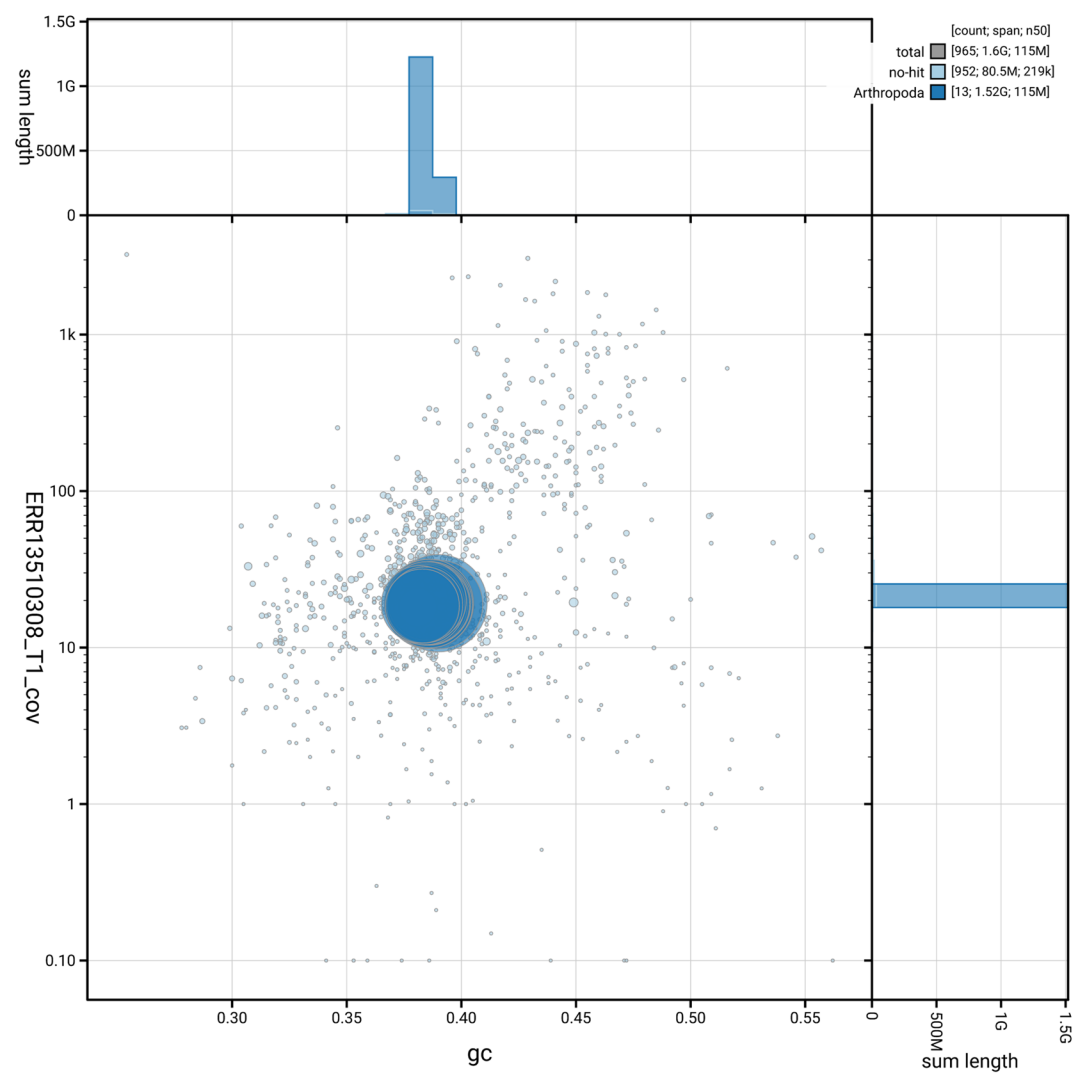
Genome assembly of *Ischnura senegalensis*, ioIscSene1.hap1.1: BlobToolKit GC-coverage plot. Blob plot showing sequence coverage (vertical axis) and GC content (horizontal axis). The circles represent scaffolds, with the size proportional to scaffold length and the colour representing phylum membership. The histograms along the axes display the total length of sequences distributed across different levels of coverage and GC content. An interactive version of this figure is available at https://blobtoolkit.genomehubs.org/view/GCA_964251815.1/blob.

**Figure 4.**
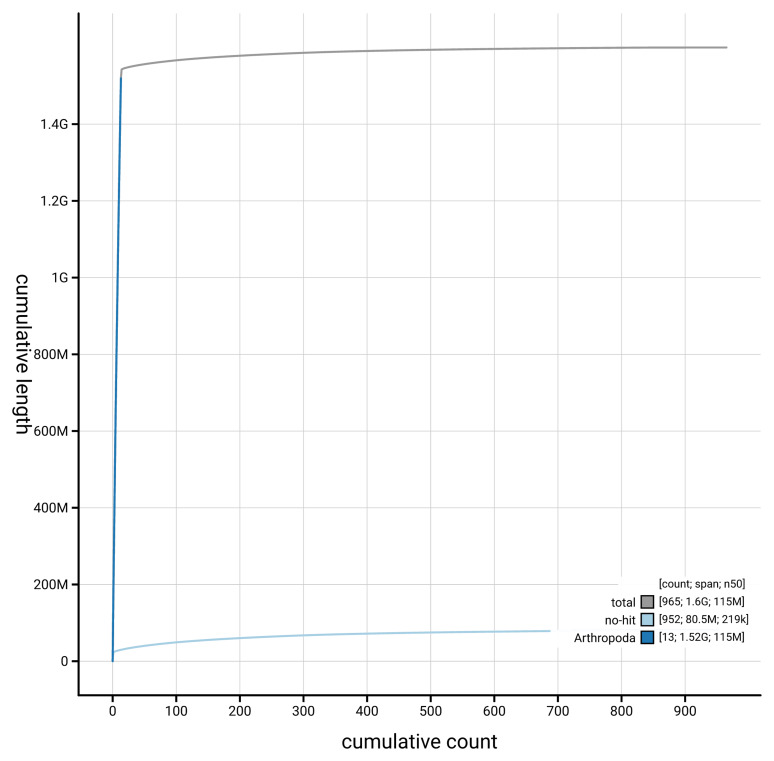
Genome assembly of *Ischnura senegalensis,* ioIscSene1.hap1.1: BlobToolKit cumulative sequence plot. The grey line shows cumulative length for all scaffolds. Coloured lines show cumulative lengths of scaffolds assigned to each phylum using the buscogenes taxrule. An interactive version of this figure is available at https://blobtoolkit.genomehubs.org/view/GCA_964251815.1/cumulative.

The snail plot in Figure 2 provides a summary of the assembly statistics, indicating the distribution of scaffold lengths and other assembly metrics. Figure 3 shows the distribution of scaffolds by GC proportion and coverage. Figure 4 presents a cumulative assembly plot, with separate curves representing different scaffold subsets assigned to various phyla, illustrating the completeness of the assembly.

**Figure 5.**
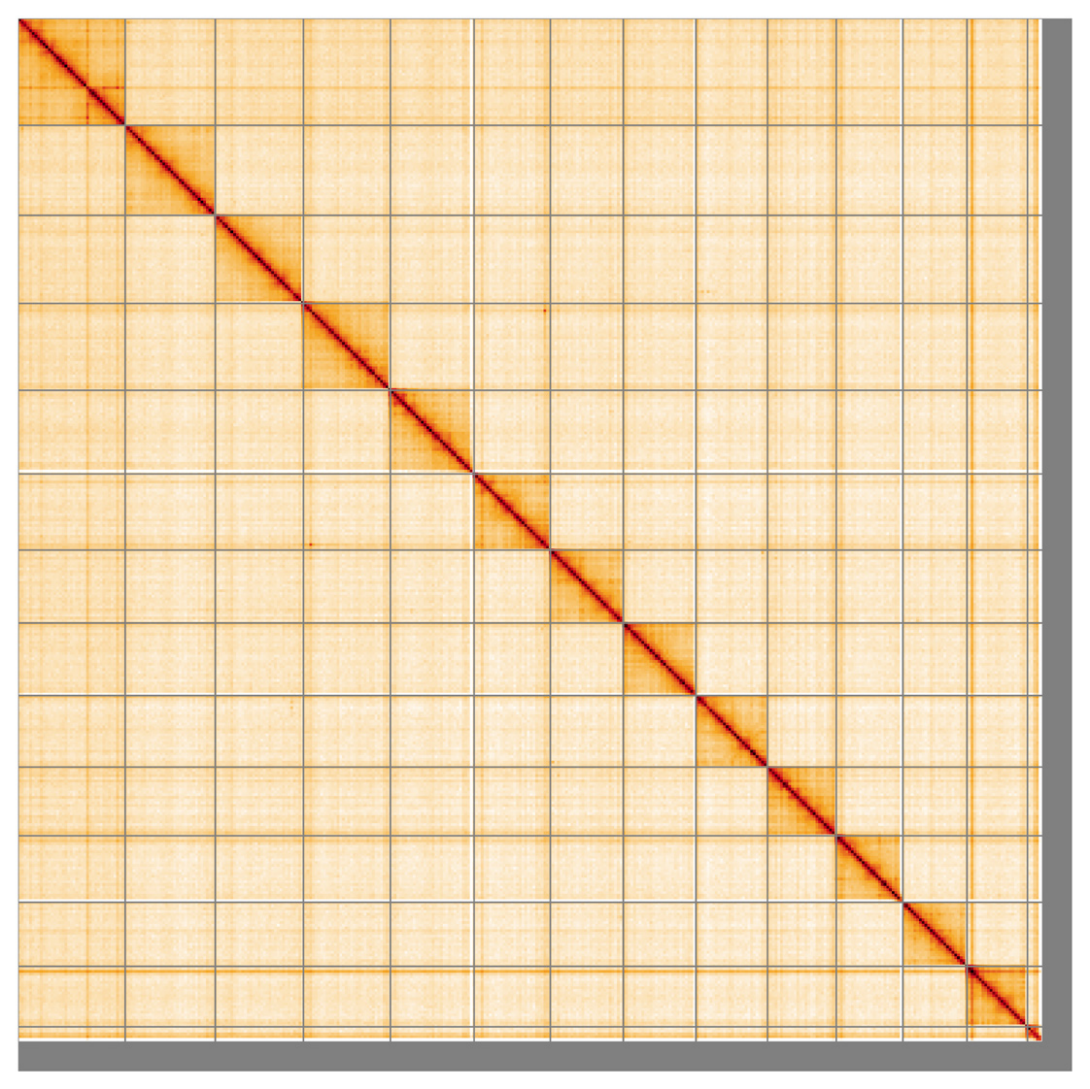
Genome assembly of *Ischnura senegalensis,* ioIscSene1.hap1.1 *:* Hi-C contact map of the ioIscSene1.hap1.1 assembly, visualised using HiGlass. Chromosomes are shown in order of size from left to right and top to bottom. An interactive version of this figure may be viewed at https://genome-note-higlass.tol.sanger.ac.uk/l/?d=ImiUWL5mQjWx_YWAMiEKFw.

Most of the assembly sequence (96.41%) was assigned to 14 chromosomal-level scaffolds, representing 13 autosomes and the X sex chromosome. These chromosome-level scaffolds, confirmed by Hi-C data, are named according to size ( Figure 5; Table 3). During curation, chromosome X was identified by homology with the assembly of *Ischnura elegans* (GCF_921293095.1) ( Price *et al*., 2022).

**Table 3.** Chromosomal pseudomolecules in the genome assembly of *Ischnura senegalensis*, ioIscSene1.hap1.1.

INSDC accession	Name	Length (Mb)	GC%
OZ176221.1	1	160.94	39
OZ176222.1	2	135.56	38.5
OZ176223.1	3	132.68	39
OZ176224.1	4	131.21	38.5
OZ176225.1	5	125.68	38.5
OZ176226.1	6	114.74	38.5
OZ176227.1	7	110.04	38.5
OZ176228.1	8	109.24	38.5
OZ176229.1	9	107.73	38.5
OZ176231.1	10	100.3	38
OZ176232.1	11	96.52	38.5
OZ176233.1	12	91.23	38.5
OZ176234.1	13	23.03	38
OZ176230.1	X	103.43	38.5
OZ176235.1	MT	0.02	26

The mitochondrial genome was also assembled. This sequence is included as a contig in the multifasta file of the genome submission and as a standalone record in GenBank.

### Assembly quality metrics

The estimated Quality Value (QV) and *k*-mer completeness metrics, along with BUSCO completeness scores, were calculated for each haplotype and the combined assembly. The QV reflects the base-level accuracy of the assembly, while *k*-mer completeness indicates the proportion of expected *k*-mers identified in the assembly. BUSCO scores provide a measure of completeness based on benchmarking universal single-copy orthologues.

For haplotype 1, the estimated QV is 61.4, and for haplotype 2, the QV is 61.3. When the two haplotypes are combined, the assembly achieves an estimated QV of 61.4. The *k*-mer completeness for haplotype 1 is 71.73%, for haplotype 2, 71.86%, and for the combined haplotypes, 99.19%. BUSCO 5.5.0 analysis using the insecta_odb10 reference set ( *n* = 1,367) achieved a completeness score of 97.0% (single = 96.2%, duplicated = 0.8%) for haplotype 1.

Table 2 provides assembly metric benchmarks adapted from Rhie *et al.* (2021) and the Earth BioGenome Project (EBP) Report on Assembly Standards September 2024. The assembly achieves the EBP reference standard of 6.C.61.

## Methods

### Sample acquisition

An adult female *Ischnura senegalensis* (specimen ID SAN20001545, ToLID ioIscSene1) was collected from Clementi Forest, Central Region (latitude 1.33, longitude 103.78) on 2022-11-18. The specimen was collected and identified by Beatriz Willink (National University of Singapore).

### Nucleic acid extraction

The workflow for high molecular weight (HMW) DNA extraction at the Wellcome Sanger Institute (WSI) Tree of Life Core Laboratory includes a sequence of procedures: sample preparation and homogenisation, DNA extraction, fragmentation and purification. Detailed protocols are available on protocols.io ( Denton *et al.*, 2023b). The ioIscSene1 sample was prepared for DNA extraction by weighing and dissecting it on dry ice ( Jay *et al.*, 2023). Tissue from the whole organism was homogenised using a PowerMasher II tissue disruptor ( Denton *et al.*, 2023a).

HMW DNA was extracted in the WSI Scientific Operations core using the Automated MagAttract v2 protocol ( Oatley *et al.*, 2023a). For ultra-low input (ULI) PacBio sequencing, DNA was fragmented using the Covaris g-TUBE method (Oatley *et al*., 2023c). Sheared DNA was purified by solid-phase reversible immobilisation, using AMPure PB beads to eliminate shorter fragments and concentrate the DNA ( Strickland *et al.*, 2023). The concentration of the sheared and purified DNA was assessed using a Nanodrop spectrophotometer and Qubit Fluorometer using the Qubit dsDNA High Sensitivity Assay kit. Fragment size distribution was evaluated by running the sample on the FemtoPulse system.

### Hi-C sample preparation

Tissue from the whole organism of the ioIscSene1 sample was processed for Hi-C sequencing at the WSI Scientific Operations core, using the Arima-HiC v2 kit. In brief, 20–50 mg of frozen tissue (stored at –80 °C) was fixed, and the DNA crosslinked using a TC buffer with 22% formaldehyde concentration. After crosslinking, the tissue was homogenised using the Diagnocine Power Masher-II and BioMasher-II tubes and pestles. Following the Arima-HiC v2 kit manufacturer's instructions, crosslinked DNA was digested using a restriction enzyme master mix. The 5’-overhangs were filled in and labelled with biotinylated nucleotides and proximally ligated. An overnight incubation was carried out for enzymes to digest remaining proteins and for crosslinks to reverse. A clean up was performed with SPRIselect beads prior to library preparation. Additionally, the biotinylation percentage was estimated using the Qubit Fluorometer v4.0 (Thermo Fisher Scientific) and Qubit HS Assay Kit and Arima-HiC v2 QC beads.

### Library preparation and sequencing

Library preparation and sequencing were performed at the WSI Scientific Operations core.

***PacBio HiFi (ULI)***

The sample requires Covaris g-TUBE shearing to approximately 10 kb prior to library preparation. Ultra-low input libraries were prepared using PacBio SMRTbell® Express Template Prep Kit 2.0 and PacBio SMRTbell® gDNA Sample Amplification Kit. To begin, samples were normalised to 20 ng of DNA. Initial removal of single-strand overhangs, DNA damage repair, and end repair/A-tailing were performed per manufacturer’s instructions. From the SMRTbell® gDNA Sample Amplification Kit, amplification adapters were then ligated. A 0.85X pre-PCR clean-up was performed with Promega ProNex beads and the sample was then divided into two for a dual PCR. PCR reactions A and B each followed the PCR programs as described in the manufacturer’s protocol. A 0.85X post-PCR clean-up was performed with ProNex beads for PCR reactions A and B and DNA concentration was quantified using the Qubit Fluorometer v4.0 (Thermo Fisher Scientific) and Qubit HS Assay Kit and fragment size analysis was carried out using the Agilent Femto Pulse Automated Pulsed Field CE Instrument (Agilent Technologies) and gDNA 55kb BAC analysis kit. PCR reactions A and B were then pooled, ensuring the total mass was ≥500 ng in 47.4 μl. The pooled sample then repeated the process for DNA damage repair, end repair/A-tailing and additional hairpin adapter ligation. A 1X clean-up was performed with ProNex beads and DNA concentration was quantified using the Qubit and fragment size analysis was carried out using the Agilent Femto Pulse Automated Pulsed Field CE Instrument (Agilent Technologies). Size selection was performed using Sage Sciences' PippinHT system with target fragment size determined by analysis from the Femto Pulse, usually a value between 4000 and 9000 bp. Size selected libraries were then cleaned-up using1.0X ProNex beads and normalised to 2 nM before proceeding to sequencing.

Samples were sequenced on a Revio instrument (Pacific Biosciences, California, USA). Prepared libraries were normalised to 2 nM, and 15 μL was used for making complexes. Primers were annealed and polymerases were hybridised to create circularised complexes according to manufacturer’s instructions. The complexes were purified with the 1.2X clean up with SMRTbell beads. The purified complexes were then diluted to the Revio loading concentration (in the range 200–300 pM), and spiked with a Revio sequencing internal control. Samples were sequenced on Revio 25M SMRT cells (Pacific Biosciences, California, USA). The SMRT link software, a PacBio web-based end-to-end workflow manager, was used to set-up and monitor the run, as well as perform primary and secondary analysis of the data upon completion.

***Hi-C***

For Hi-C library preparation, DNA was fragmented using the Covaris E220 sonicator (Covaris) and size selected using SPRISelect beads to 400 to 600 bp. The DNA was then enriched using the Arima-HiC v2 kit Enrichment beads. Using the NEBNext Ultra II DNA Library Prep Kit (New England Biolabs) for end repair, a-tailing, and adapter ligation. This uses a custom protocol which resembles the standard NEBNext Ultra II DNA Library Prep protocol but where library preparation occurs while DNA is bound to the Enrichment beads. For library amplification, 10 to 16 PCR cycles were required, determined by the sample biotinylation percentage. The Hi-C sequencing was performed using paired-end sequencing with a read length of 150 bp on an Illumina NovaSeq X instrument.

### Genome assembly, curation and evaluation

***Assembly***

Prior to assembly of the PacBio HiFi reads, a database of *k*-mer counts ( *k* = 31) was generated from the filtered reads using FastK. GenomeScope2 ( Ranallo-Benavidez *et al.*, 2020) was used to analyse the *k*-mer frequency distributions, providing estimates of genome size, heterozygosity, and repeat content.

The HiFi reads were assembled using Hifiasm in Hi-C phasing mode ( Cheng *et al.*, 2021; Cheng *et al.*, 2022), resulting in a pair of haplotype-resolved assemblies. The Hi-C reads were mapped to the primary contigs using bwa-mem2 ( Vasimuddin *et al.*, 2019). The contigs were further scaffolded using the provided Hi-C data ( Rao *et al.*, 2014) in YaHS ( Zhou *et al.*, 2023) using the --break option for handling potential misassemblies. The scaffolded assemblies were evaluated using Gfastats ( Formenti *et al.*, 2022), BUSCO ( Manni *et al.*, 2021) and MERQURY.FK ( Rhie *et al.*, 2020).

The mitochondrial genome was assembled using MitoHiFi ( Uliano-Silva *et al.*, 2023), which runs MitoFinder ( Allio *et al.*, 2020) and uses these annotations to select the final mitochondrial contig and to ensure the general quality of the sequence.

***Assembly curation***

The assembly was decontaminated using the Assembly Screen for Cobionts and Contaminants (ASCC) pipeline (article in preparation). Flat files and maps used in curation were generated in TreeVal ( Pointon *et al.*, 2023). Manual curation was primarily conducted using PretextView ( Harry, 2022), with additional insights provided by JBrowse2 ( Diesh *et al.*, 2023) and HiGlass ( Kerpedjiev *et al.*, 2018). Scaffolds were visually inspected and corrected as described by Howe *et al*. (2021). Any identified contamination, missed joins, and mis-joins were corrected, and duplicate sequences were tagged and removed. Sex chromosomes were identified by synteny analysis. The curation process is documented at https://gitlab.com/wtsi-grit/rapid-curation (article in preparation).

***Assembly quality assessment***

The Merqury.FK tool ( Rhie *et al.*, 2020), run in a Singularity container ( Kurtzer *et al.*, 2017), was used to evaluate *k*-mer completeness and assembly quality for the primary and alternate haplotypes using the *k*-mer databases ( *k* = 31) that were computed prior to genome assembly. The analysis outputs included assembly QV scores and completeness statistics.

A Hi-C contact map was produced for the final version of the assembly. The Hi-C reads were aligned using bwa-mem2 ( Vasimuddin *et al.*, 2019) and the alignment files were combined using SAMtools ( Danecek *et al.*, 2021). The Hi-C alignments were converted into a contact map using BEDTools ( Quinlan & Hall, 2010) and the Cooler tool suite ( Abdennur & Mirny, 2020). The contact map is visualised in HiGlass ( Kerpedjiev *et al.*, 2018).

The blobtoolkit pipeline is a Nextflow port of the previous Snakemake Blobtoolkit pipeline ( Challis *et al.*, 2020). It aligns the PacBio reads in SAMtools and minimap2 ( Li, 2018) and generates coverage tracks for regions of fixed size. In parallel, it queries the GoaT database ( Challis *et al.*, 2023) to identify all matching BUSCO lineages to run BUSCO ( Manni *et al.*, 2021). For the three domain-level BUSCO lineages, the pipeline aligns the BUSCO genes to the UniProt Reference Proteomes database ( Bateman *et al.*, 2023) with DIAMOND blastp ( Buchfink *et al.*, 2021). The genome is also divided into chunks according to the density of the BUSCO genes from the closest taxonomic lineage, and each chunk is aligned to the UniProt Reference Proteomes database using DIAMOND blastx. Genome sequences without a hit are chunked using seqtk and aligned to the NT database with blastn ( Altschul *et al.*, 1990). The blobtools suite combines all these outputs into a blobdir for visualisation.

The blobtoolkit pipeline was developed using nf-core tooling ( Ewels *et al.*, 2020) and MultiQC ( Ewels *et al.*, 2016), relying on the Conda package manager, the Bioconda initiative ( Grüning *et al.*, 2018), the Biocontainers infrastructure ( da Veiga Leprevost *et al.*, 2017), as well as the Docker ( Merkel, 2014) and Singularity ( Kurtzer *et al.*, 2017) containerisation solutions.

Table 4 contains a list of relevant software tool versions and sources.

**Table 4.** Software tools: versions and sources.

Software tool	Version	Source
BEDTools	2.30.0	https://github.com/arq5x/bedtools2
BLAST	2.14.0	ftp://ftp.ncbi.nlm.nih.gov/blast/executables/blast+/
BlobToolKit	4.3.9	https://github.com/blobtoolkit/blobtoolkit
BUSCO	5.5.0	https://gitlab.com/ezlab/busco
bwa-mem2	2.2.1	https://github.com/bwa-mem2/bwa-mem2
Cooler	0.8.11	https://github.com/open2c/cooler
DIAMOND	2.1.8	https://github.com/bbuchfink/diamond
fasta_windows	0.2.4	https://github.com/tolkit/fasta_windows
FastK	427104ea91c78c3b8b8b49f1a7d6bbeaa869ba1c	https://github.com/thegenemyers/FASTK
Gfastats	1.3.6	https://github.com/vgl-hub/gfastats
GoaT CLI	0.2.5	https://github.com/genomehubs/goat-cli
Hifiasm	0.19.8-r603	https://github.com/chhylp123/hifiasm
HiGlass	44086069ee7d4d3f6f3f0012569789ec138f42b84 aa44357826c0b6753eb28de	https://github.com/higlass/higlass
MerquryFK	d00d98157618f4e8d1a9190026b19b471055b22e	https://github.com/thegenemyers/MERQURY.FK
Minimap2	2.24-r1122	https://github.com/lh3/minimap2
MitoHiFi	3	https://github.com/marcelauliano/MitoHiFi
MultiQC	1.14, 1.17, and 1.18	https://github.com/MultiQC/MultiQC
NCBI Datasets	15.12.0	https://github.com/ncbi/datasets
Nextflow	23.10.0	https://github.com/nextflow-io/nextflow
PretextView	0.2.5	https://github.com/sanger-tol/PretextView
samtools	1.19.2	https://github.com/samtools/samtools
sanger-tol/ascc	-	https://github.com/sanger-tol/ascc
sanger-tol/blobtoolkit	0.5.1	https://github.com/sanger-tol/blobtoolkit
Seqtk	1.3	https://github.com/lh3/seqtk
Singularity	3.9.0	https://github.com/sylabs/singularity
TreeVal	1.2.0	https://github.com/sanger-tol/treeval
YaHS	1.2a.2	https://github.com/c-zhou/yahs

### Wellcome Sanger Institute – Legal and Governance

The materials that have contributed to this genome note have been supplied by a Tree of Life collaborator.

The Wellcome Sanger Institute employs a process whereby due diligence is carried out proportionate to the nature of the materials themselves, and the circumstances under which they have been/are to be collected and provided for use. The purpose of this is to address and mitigate any potential legal and/or ethical implications of receipt and use of the materials as part of the research project, and to ensure that in doing so we align with best practice wherever possible.

The overarching areas of consideration are:

- Ethical review of provenance and sourcing of the material
- Legality of collection, transfer and use (national and international)

Each transfer of samples is undertaken according to a Research Collaboration Agreement or Material Transfer Agreement entered into by the Tree of Life collaborator, Genome Research Limited (operating as the Wellcome Sanger Institute) and in some circumstances other Tree of Life collaborators.

## Data Availability

European Nucleotide Archive: Ischnura senegalensis (tropical bluetail). Accession number PRJEB78898; https://identifiers.org/ena.embl/PRJEB78898. The genome sequence is released openly for reuse by the Wellcome Sanger Institute Tree of Life Programme. All raw sequence data and the assembly have been deposited in INSDC databases. The genome will be annotated using available RNA-Seq data and presented through the Ensembl pipeline at the European Bioinformatics Institute. Raw data and assembly accession identifiers are reported in Table 1 and Table 2.

## Author information

Members of the Wellcome Sanger Institute Tree of Life Management, Samples and Laboratory team are listed here: https://doi.org/10.5281/zenodo.12162482.

Members of Wellcome Sanger Institute Scientific Operations: Sequencing Operations are listed here: https://doi.org/10.5281/zenodo.12165051.

Members of the Wellcome Sanger Institute Tree of Life Core Informatics team are listed here: https://doi.org/10.5281/zenodo.12160324.

Members of the Tree of Life Core Informatics collective are listed here: https://doi.org/10.5281/zenodo.12205391.
