# Supplementary material for: The genome sequence of the Tropical Bluetail Damselfly, Ischnura senegalensis (Rambur, 1842)
Source: Wellcome Open Res. 2025 Feb 24;10:104. [Version 1] doi: 10.12688/wellcomeopenres.23747.1 (PMC12033981; doi:10.12688/wellcomeopenres.23747.1)
Supplement: Peer review history

# Reviewer report: Reviewer response for version 1

**DOI:** 10.21956/wellcomeopenres.26195.r121661

**Published:** 2025-04-26

**Authors:** Merly Escalona

**Affiliations:** 1 University of California Santa Cruz, Santa Cruz, California, USA

Willink et al. described the generation of resources for the Tropical Bluetail Damselfly, along with a relevant justification for their creation.

 The generated resources include a diploid genome assembly and a mitochondrial genome assembly. The nuclear genome assembly meets the metrics established for high-quality genome assemblies, as presented in Rhie et al. (2021).

 The methods used to create such resources are robust, and sequencing data and generated resources have been made available. 

 A minor comment I have is related to the minimum quality metrics generated for the mitochondrial assembly.

Are sufficient details of methods and materials provided to allow replication by others?

Yes

Is the rationale for creating the dataset(s) clearly described?

Yes

Are the datasets clearly presented in a useable and accessible format?

Yes

Are the protocols appropriate and is the work technically sound?

Yes

Reviewer Expertise:

bioinformatics and genome assembly

I confirm that I have read this submission and believe that I have an appropriate level of expertise to confirm that it is of an acceptable scientific standard.

# Reviewer report: Reviewer response for version 1

**DOI:** 10.21956/wellcomeopenres.26195.r120175

**Published:** 2025-03-07

**Authors:** Jason Charamis

**Affiliations:** 1 Foundation for Research and Technology - Hellas, Irákleion, Greece

This manuscript presents a valuable contribution to the field with the genome sequencing and assembly of the Tropical Bluetail Damselfly, *Ischnura senegalensis*. The technical aspects of the work are robust, with appropriate methodological choices for sequencing and assembly that have yielded a high-quality genome.

 The authors provide compelling justification for the importance of this species in the broader context of Odonata genomics. Particularly noteworthy is their detailed explanation of how this genome will facilitate comparative genomic analyses of evolutionary innovations specific to this order, particularly morph differentiation.

**I only have four minor comments:**

 1. (NCBI:txid126660) should be (NCBI: txid126660)

 2. References for the nice citations in the Background do not exist - maybe this has something to do with the citation manager. 

 3. (Willink *et al*., 2024b) in the first sentence of Background should become (Willink *et al*., 2024a)

 4. Next occasions of (Willink *et al*., 2024a) should be come (Willink *et al*., 2024b)

Are sufficient details of methods and materials provided to allow replication by others?

Yes

Is the rationale for creating the dataset(s) clearly described?

Yes

Are the datasets clearly presented in a useable and accessible format?

Yes

Are the protocols appropriate and is the work technically sound?

Yes

Reviewer Expertise:

Arthropod Comparative Genomics

I confirm that I have read this submission and believe that I have an appropriate level of expertise to confirm that it is of an acceptable scientific standard.
